# Regression analysis for the determination of microplastics in sediments using differential scanning calorimetry

**DOI:** 10.1007/s11356-024-33100-8

**Published:** 2024-04-15

**Authors:** Sven Schirrmeister, Lucas Kurzweg, Xhoen Gjashta, Martin Socher, Andreas Fery, Kathrin Harre

**Affiliations:** 1 Faculty of Agriculture, Environment and Chemistry, University of Applied Sciences Dresden, Friedrich-List-Platz 1, 01069 Dresden, Germany; 2 Leibniz Institut für Polymerforschung Dresden e.V., Institute for Physical Chemistry and Polymer Physics, Hohe Str. 6, 01069 Dresden, Germany; 3 Faculty of Chemistry and Food Chemistry, Division of Physical Chemistry of Polymeric Materials, Technical University Dresden, Mommsenstraße 6, 01069 Dresden, Germany

**Keywords:** Microplastic, Polymers, Sediment, Thermal analysis, DSC, Regression

## Abstract

This research addresses the growing need for fast and cost-efficient methods for microplastic (MP) analysis. We present a thermo-analytical method that enables the identification and quantification of different polymer types in sediment and sand composite samples based on their phase transition behavior. Differential scanning calorimetry (DSC) was performed, and the results were evaluated by using different regression models. The melting and crystallization enthalpies or the change in heat capacity at the glass transition point were measured as regression analysis data. Ten milligrams of sea sand was spiked with 0.05 to 1.5 mg of microplastic particles (size: 100 to 200 µm) of the semi-crystalline polymers LD-PE, HD-PE, PP, PA6, and PET, and the amorphous polymers PS and PVC. The results showed that a two-factorial regression enabled the unambiguous identification and robust quantification of different polymer types. The limits of quantification were 0.13 to 0.33 mg and 0.40 to 1.84 mg per measurement for semi-crystalline and amorphous polymers, respectively. Moreover, DSC is robust with regard to natural organic matrices and allows the fast and non-destructive analysis of microplastic within the analytical limits. Hence, DSC could expand the range of analytical methods for microplastics and compete with perturbation-prone chemical analyses such as thermal extraction–desorption gas chromatography–mass spectrometry or spectroscopic methods. Further work should focus on potential changes in phase transition behavior in more complex matrices and the application of DSC for MP analysis in environmental samples.

## Introduction

Microplastic (MP) is described as polymer particles with diameters of less than 5 mm, including differently shaped particles as well as fibers. Pollution due to MP is considered to be ubiquitous (Browne et al. 2011, Akdogan and Guven 2019). Several studies have detected MP in various compartments of the environment such as the atmosphere (Kernchen et al. 2021), rivers (Klein et al. 2015; Skalska et al. 2020; Meijer et al. 2021), shores (Browne et al. 2011), the deep sea (van Cauwenberghe et al. 2013), Arctic ice (Peeken et al. 2018), and the highest mountains (Napper et al. 2020). Moreover, the amount of polymeric material in the oceans might double by 2050 (Lebreton et al. 2019). Adverse effects on plants (Souza Machado et al. 2019) and microorganisms, such as *Daphnia magna* (An et al. 2021), have already been demonstrated. The human health effects have not yet been adequately studied (Issac and Kandasubramanian 2021), but there are already indications and suggestions for future research (Wieland et al. 2022). Thus, legislation is already responding to the increasing environmental impact of microplastics for example, in the EU directive on the quality of water intended for human consumption, where microplastics are included on the watch list (European Parliament 2023). In addition, the legislation stipulates that an analysis is required by 2024 to enable a risk assessment for microplastics. Accordingly, a significant increase in the prevalence of MP analysis is expected in the coming years, with a strong focus on reliable, precise, fast, and cost-efficient methods.

At present, there is no standardized protocol for MP analysis, leading to the application of different methods for their identification and quantification, which are only comparable to a limited extent. However, comprehensive routine analysis and harmonization are urgently needed to understand the pathways, distribution, and impacts of MP (Shahul Hamid et al. 2018; Brander et al. 2020). The accurate determination of MP is rather challenging because of the diversity of polymeric materials. The molecular compositions of plastics and their modifications are very complex. However, it has been shown that the most common polymers in environmental samples are polyethylene (PE), polypropylene (PP), polyamide (PA), polyethylene terephthalate (PET), polystyrene (PS), and polyvinyl chloride (PVC), on which MP analysis may focus (Way et al. 2022; Yang et al. 2021). In total, these polymers constitute more than 70% of the globally produced plastics (Plastics Europe 2023).

The particle sizes of MP are comparable to the particle sizes of sediments. The challenge of MP determination in the environment lies in the similarity of natural organic material and MP from a chemical analytical point of view. In addition, an analysis can determine either the mass of MP or the number of MP particles in a particulate sample matrix. Generally, two different analytical approaches have been established so far for the analysis of MP. The first comprises spectroscopic methods (Fourier-transform infrared (FTIR) and Raman spectroscopy), which determine the number of MP particles per sample volume. However, such methods for the determination of the number of particles can only be applied after complex and time-consuming sample preparation procedures. The second approach comprises thermo-analytical methods (Peñalver et al. 2020). These methods provide mass-specific information (Waldman and Rillig 2020; Perez et al. 2022; Haines 2002), such as the mass of polymer per mass of the sample or per volume fluid medium. The information about the size and shape of the MP particles is lost in mass determination methods and cannot be detected. Currently, the establishment of thermal extraction–desorption gas chromatography-mass spectrometry (TED-GC–MS) as a thermo-analytical method is being pursued (Goedecke et al. 2020). This method combines thermo-gravimetry and the chemical analysis of the thermal degradation products of microplastic particles in environmental samples, and it is rather robust to matrix-related influences (Goedecke et al. 2020). Nevertheless, TED-GC–MS devices are expensive and require a large investment to realize comprehensive MP monitoring. Therefore, the focus of this work is the application of differential scanning calorimetry (DSC) as a fast and cost-efficient method for MP analysis. DSC is a technique used for the detection and analysis of thermal transitions in various types of polymers, including crystalline, semi-crystalline, and amorphous polymers. DSC measures the heat flow into or out of a sample as a function of the temperature, allowing the observation of events such as melting, crystallization, or glass transition. These transitions provide valuable insights into the physical properties and polymer types. DSC analysis has already been compared in inter-laboratory tests (Becker et al. 2020), and its suitability for the determination of microplastics has already been described (Bitter and Lackner 2021; Shabaka and Ghobashy 2019). An important advantage of DSC is the high robustness of this method against different matrices, which typically display organic contamination from environmental samples. Organic contaminants, which have chemically similar structures to the monomer units in polymers (e.g., esters, olefins, amino acids) do not show such phase transformations and therefore cannot be detected by DSC measurements. Hence, the purification of samples by using chemical or enzymatic methods is not required, and this minimizes the analytical effort by reducing the number of steps performed. Moreover, DSC can be considered a non-destructive method for the chemical structure of the polymer within certain temperature limits. Until the temperature at which the thermal degradation of a polymer begins, the chemical information about the polymer is preserved during a DSC measurement. However, at temperatures above the onset of thermal degradation, DSC is a destructive method. Therefore, the limitation of DSC as a chemically non-destructive method is the polymer-specific thermal degradation point; however, this is not reached in the procedure applied in this study. This enables the confirmation of polymer type with other methods like microscopic-spectroscopic methods.

Polymer detection by DSC is based on the degree of crystallinity or the change in the internal volume of the polymer in microplastic particles. All thermodynamic processes, such as melting or crystallization, are characterized by a melting and crystallization peak associated with each polymer. In connection with thermal degradation, a fingerprint is described in the literature (Wunderlich 2005) that allows the identification of different polymer types. The same applies to the glass transition point. As with all thermo-analytical methods, DSC requires calibration with external standards to determine the MP mass in a sample. Our study focuses on the evaluation of different regression models to enable DSC for MP determination. This paper describes the statistical evaluation of DSC for the identification and quantification of microplastics in particulate matrices with respect to the robustness of the limits of detection and quantification. In addition to semi-crystalline commodity polymers, namely LD-PE, HD-PE, PP, PA 6, and PET, the amorphous polymers PS and PVC are included in this study. A linear regression and a two-factorial regression based on the melting and crystallization enthalpies or the change in the specific heat capacity at the point of glass transition are compared. In addition, polycaprolactone (PCL) is discussed as a possible internal standard for environmental samples.

## Methods

### Materials and sample preparation

MP particles were obtained via the cryomilling (Pulverisette 0, Fritsch, Idar-Oberstein, Germany, WC *r* = 50 mm, amplitude = 1.5 mm, liquid nitrogen) of eight different polymers (Table 1). The particles were sieved to a particle size of 100–200 µm using a vibratory sieve shaker (ANALYSETTE 3, Fritsch, Idar-Oberstein, Germany) equipped with stainless-steel sieves from Fritsch. Sea sand was used as an inorganic matrix to produce mixtures with the prepared MP particles. The sea sand was sieved to a particle size of 100–200 µm as well but without milling.

**Table 1:** List of all used materials for sample preparation, including sea sand and polymers, and conditions of DSC measurements

Material	Supplier // product	Range of polymer mass (mg)	DSC-program
Sea sand	VWR.com // commercial product	-	I, II
LD-PE	LyondellBasell Industries // Lupolen 2420K	0.05–1.5	I
HD-PE	Sigmal-Aldrich // MKCL3235	0.05–1.5	I
PP	LyondellBasell Industries // Moplen EF300N copolymer	0.05–1.5	I
PA 6	BASF // Ultramid B3S	0.05–1.5	I
PS	BASF // Polystyrol 158K	1.0–3.25	I
PET	Kruschitz // KRUPET-A IV 0,7–0,8	0.1–1.5	I
PCL	Abifor // #16010/8/Typ1639	0.05–1.5	I
PVC	AlphaGary // 2228C-50S Clear 0003 UV	0.1–1.5	II

The samples were prepared by weighing 10 ± 1 mg of sea sand into a DSC crucible (Concavus 40 µl, Netzsch, Selb, Germany) and then the corresponding mass of the polymer to an accuracy level of ± 0.05 mg. The laboratory balance (XSR DU 105, Mettler Toledo, Giessen, Germany) had a certified level of tolerance of ± 0.03 mg.

The lowest polymer content was 0.05 mg per sample, following the values of 0.10, 0.15, 0.20, 0.25, 0.50, 0.75, 1.00, and 1.25 mg, and the highest polymer content was 1.50 mg per sample. For PS, the values were 1.00, 1.25, 1.50, 1.75, 2.00, 2.25, 2.50, 2.75, 3.00, and 3.25 mg. Three replicas were measured for each polymer content value. Accordingly, a total of 30 samples were prepared for each polymer, as shown in Table 1. Each crucible was closed, and the lid was pierced with a steel needle. Each sample was prepared by weighing the sand directly in the crucible and then adding the polymer on top of the sand.

### DSC measurements

The DSC measurements were conducted with the DSC Polyma 214 (Netzsch, Selb, Germany). The device was calibrated using the 6-reference kit produced by Netzsch. All applied temperature programs included an initial heating cycle, followed by a cooling cycle and a second heating cycle with a rate of 20 K/min. An isothermal phase of 3 min was set between temperature gradients. Cooling was performed to − 50 °C, and heating was performed to 300 °C in program (I), and to 130 °C in program (II).

**Fig. 1:**
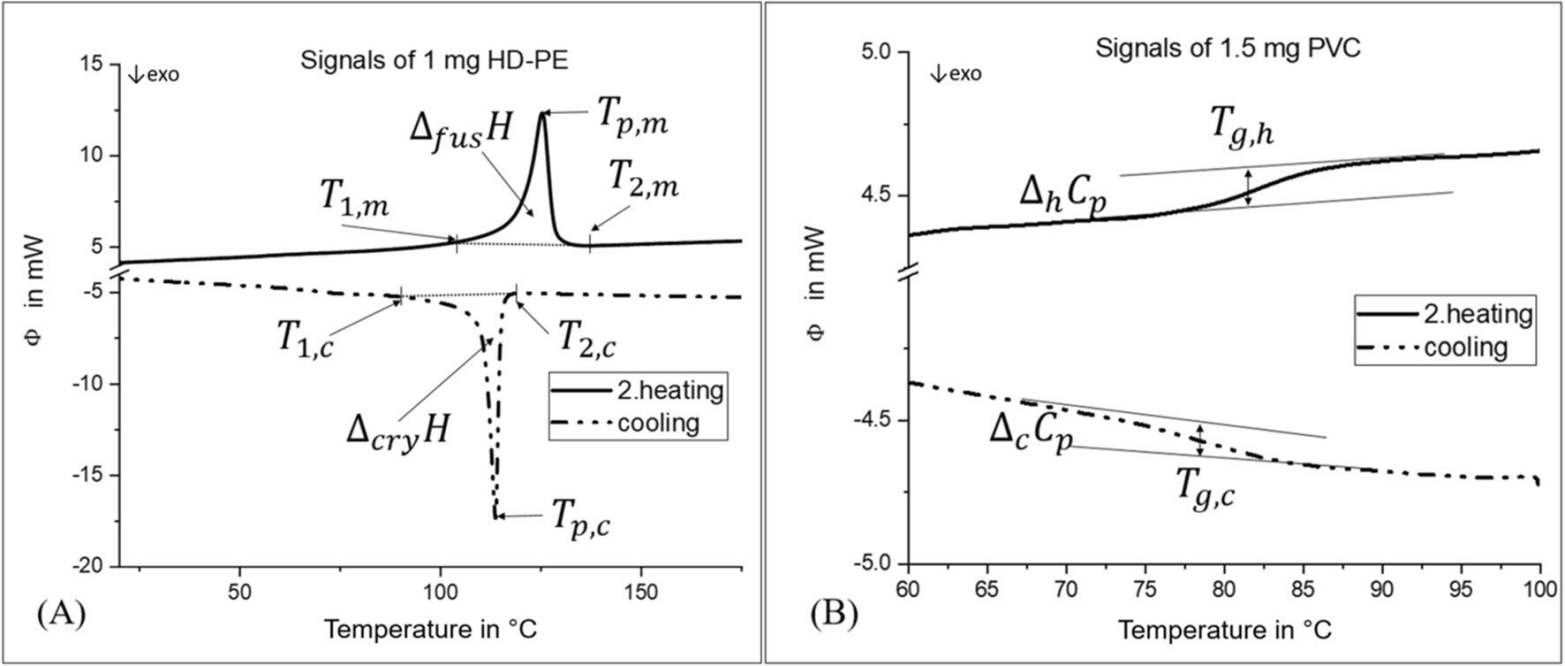
Characteristic features of thermograms for **A** semi-crystalline polymers and **B** amorphous polymers. The features can be used as regressors in linear and multiple regression models

Figure 1 shows the characteristic features of a DSC signal, which were determined from the recorded thermograms. The evaluation of the signals of the second heating cycle and of the cooling phase was conducted with the software Proteus (version 8.0.2, Netzsch, Selb, Germany). The automatic peak recognition feature of the software was used for the identification of the peak temperature (${T}_{p,m}$ and ${T}_{p,c}$).

**Fig. 2:**
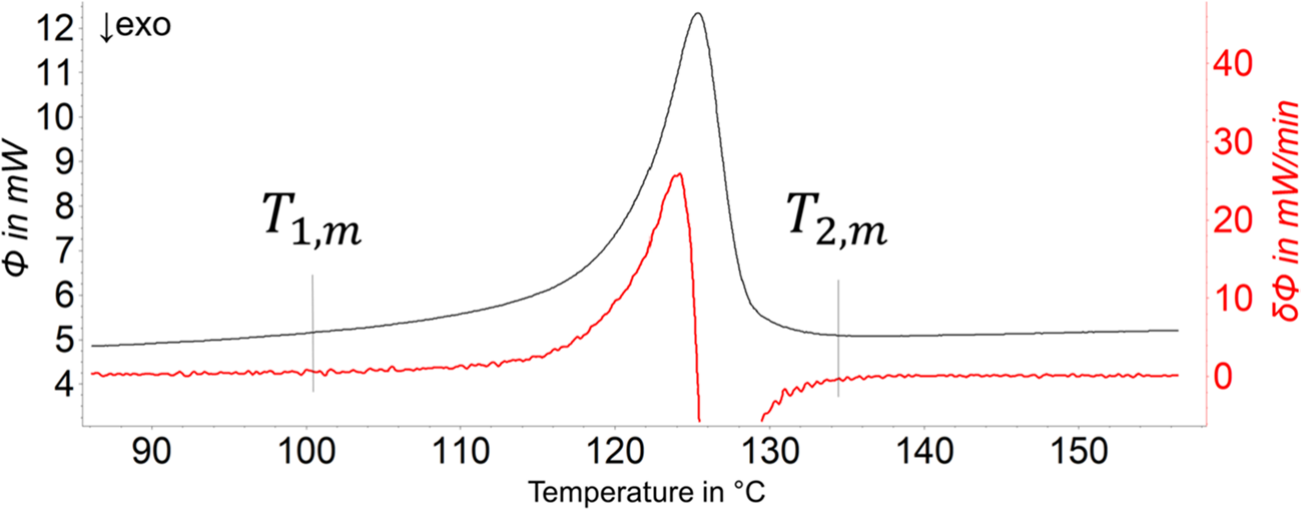
Determination of integration limits (*T*_1,m_ and *T*_2,m_) for semi-crystalline polymers. Here, an example of the determination of the melting peak area of HD-PE is shown. The black line is the DSC signal, and the red line is the first derivation of the DSC signal. The temperature at which the slope of the first derivation starts to increase is set as the lower integration limit (*T*_1,m_). The temperature at which the slope of the first derivation returns to zero is set as the upper integration limit (*T*_2,m_)

The quantitative analysis of the melting and crystallization signals was performed using a linear baseline and integration limits fitted by using the first derivation of the curve (Fig. 2). In the case of the semi-crystalline polymers, the onset (${T}_{1,m}$) and offset temperature (${T}_{2,m}$) of the melting range and the crystallization range (${T}_{1,c}$,${T}_{2,c}$) were determined from the measured data. By setting the integration limits according to the onset and offset temperatures, the melting enthalpy (${\Delta }_{{\text{fus}}}H$) and the crystallization enthalpy $({\Delta }_{{\text{cry}}}H)$, the corresponding melting peak (${T}_{p,m}$) and the crystallization peak (${T}_{p,c}$) were calculated.

**Fig. 3:**
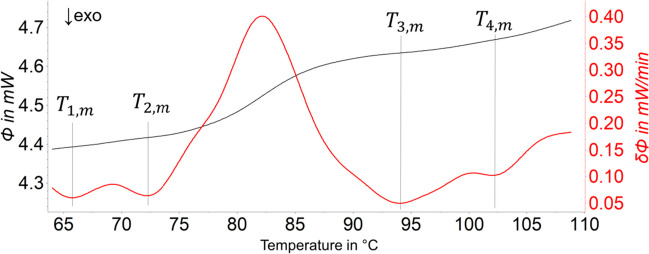
Determination of four temperatures (${T}_{1,m},{T}_{2,m}$,${T}_{3,m},$ and ${T}_{4,m}$) to enable the performance of the stepwise method for amorphous polymers. Here, an example of the determination of the *T*_g,h_ of PVC is shown. The black line is the DSC signal, and the red line is the first derivation of the DSC signal. The temperatures ${T}_{1,m}$ and ${T}_{2,m}$ were set in front of the *T*_g_ so that the baseline of the DSC signal was extended beyond the *T*_g_, which was the most suitable method. This was achieved by selecting two points on the first derivation curve with similar $\delta \phi$ values to a linear baseline. The same was applied to determine the temperatures ${T}_{3,m}$ and ${T}_{4,m}$

For amorphous polymers, the evaluation of glass transitions was performed by using the stepwise method with four temperatures (${T}_{1,m},{T}_{2,m}$,${T}_{3,m}$,${T}_{4,m}$). The determination of T_g_ referred to the midpoint temperature of the DIN EN ISO (n.d) 11357–2 standard. The four temperatures obtained based on the first deviation of the measured curve are shown in Fig. 3. As a result, the glass transitions on heating (${T}_{g,h}$) and cooling (${T}_{g,c}$) and the corresponding heat capacity changes on heating (${\Delta }_{h}{C}_{p}$) and cooling (${\Delta }_{c}{C}_{p}$) were calculated by the software.

Subsequently, the correlation of the polymer mass (*m*_i_) to the specific phase transition enthalpy ${\Delta }_{{\text{fus}}}H$ and ${\Delta }_{{\text{cry}}}H$) or the change in the specific heat capacity (${\Delta }_{h}{C}_{p}$ and ${\Delta }_{c}{C}_{p}$) was investigated with linear and multiple regression.

### Linear regression

The linear regression can be expressed in Eq. (1) for semi-crystalline and Eq. (2) for amorphous polymers. The common least squares regression method was used. The melting enthalpy (${\Delta }_{{\text{fus}}}H$) was used to calculate the mass of the semi-crystalline polymers *m*_i,sc_ with ${b}_{sc}$ as the slope and ${n}_{sc}$ as the intercept of the regression line. The mass of the amorphous polymers (*m*_i,a_) was calculated using the change in specific heat capacity during heating ${\Delta }_{h}{C}_{p}$.

1 $${m}_{i,sc}={b}_{sc}\cdot {\Delta }_{{\text{fus}}}H+{n}_{sc}$$

2 $${m}_{i,a}={b}_{a}\cdot {\Delta }_{h}{C}_{p}+{n}_{a}$$

The data from 20 samples were used as model data for the regression, and 10 samples were used as validation data to test the model (“Calculation of residuals and analytical limits” section).

### Multiple regression

In addition to the linear regression, a multiple regression model was tested. All calculations were performed using R Studio 2022.02.2 Build 443. The evaluation of the parameters shown in Fig. 1 for the determination of the mass was performed using the package “leaps” (Fahrmeir et al. 2009). As with linear regression, the software calculates a straight line with the aim of minimizing the distances to the individual measurement points (least squares method). The multiple regression in the model was based on two regressors. For semi-crystalline polymers, the regressors for the regression were the melting enthalpy ${(\Delta }_{{\text{fus}}}H$) and crystallization enthalpy (${\Delta }_{{\text{cry}}}H$), resulting in Eq. (3). For amorphous polymers, they were the changes in the specific heat capacity of the cooling (${\Delta }_{c}{C}_{p}$) and second heating cycle (${\Delta }_{h}{C}_{p}$), as indicated by Eq. (4). The regression was used to determine the polymer-specific parameters ${{a}{\prime}}_{1,sc}$ and ${{a}{\prime}}_{2,sc}$ for semi-crystalline polymers or ${{a}{\prime}}_{1,a}$ and ${{a}{\prime}}_{2,a}$ for amorphous polymers. Similar to the linear regression, an intercept ${n}{\prime}{}_{sc}$ or ${n}{\prime}{}_{a}$ was considered for semi-crystalline and amorphous polymers, respectively.

3 $${{m}^{\prime}}_{i,sc}={{a}^{\prime}}_{1,sc}\cdot {\Delta }_{{\text{fus}}}H+{{a}^{\prime}}_{2,sc}\cdot {\Delta }_{{\text{cry}}}H+{{n}^{\prime}}_{sc}$$

4 $${{m}^{\prime}}_{i,a}={{a}^{\prime}}_{1,a}\cdot {\Delta }_{h}{C}_{p}+{{a}^{\prime}}_{2,a}\cdot {\Delta }_{c}{C}_{p}+{{n}^{\prime}}_{a}$$

The data from 20 samples were used as model data. The resulting models were tested on the validation samples (*n* = 10).

### Calculation of residuals and analytical limits

As mentioned earlier, the least squares method was used for the regression models. To test the models, the residual ($${d}_{{\text{i}},{\text{x}}}$$) was calculated as the value of the difference in the MP mass predicted by the model and the actual MP mass in the sample for linear regression (Eq. (5.1)) and for multiple regression (Eq. (5.2)). In detail, the mass of the polymer in the validation samples ($${m}_{i,x};{{m}^{\prime}}_{i,x} \quad with \quad x=\left\{sc;a\right\}$$) was predicted through the regression of the model data. Subsequently, the calculated mass of the polymer in the validation samples ($${m}_{i,x}$$) was compared with the initially weighed mass ($${m}_{i,x}^{0})$$. This mass deviation in each validation sample was described as the residue ($${d}_{{\text{i}},{\text{x}}}$$). The absolute value of the maximum residue ($${d}_{i,max}$$) was used as the uncertainty parameter in this study (Eq. (6))

5.1 $${d}_{{{i}},{{x}}}={m}_{i,x}-{m}_{i,x}^{0}$$

5.2 $${{{d}}}_{{\text{i}},{\text{x}}}={{m}^{\prime}}_{i,x}-{m}_{i,x}^{0}$$

6 $${d}_{i,max}=\left|{{max}} \left\{{d}_{i,x}\right\}\right|$$

The parameter ${d}_{i,max}$ was considered the largest error. Thus, the uncertainty was assumed to be $\pm {d}_{i,max}$ for the determination of the polymer mass by DSC. The parameter ${d}_{max}$ was therefore used as the limit of detection (LOD). The limit of quantification (LOQ) was calculated according to Eq. (7) and was three times the LOD.

7 $$LOQ=3\cdot LOD=3\cdot {d}_{i,max}$$

The uncertainty in the determination of the temperature for the melting peak (*T*_p,m_), the crystallization peak (*T*_p,c_), and the glass transition (*T*_g_) was calculated on the basis of the largest deviation (± Δ*T*_x, max_) from the mean value $\widehat{{T}_{x}}$. Equation (8) was used for this calculation.

8 $$\pm\Delta {T}_{x,max}=max\left|\widehat{{T}_{x}}-{T}_{x}\right| ;x=\{p,m;\space p,c;\space g\}$$

To evaluate the regression analysis, the sum of squared residuals (SSR) and the Akaike (AIC.c) and Bayesian (BIC) information criteria were calculated from the validation data. The parameters SSR (9), AICc (10) (Hedderich and Sachs 2018), and BIC (11) (Fahrmeir et al. 2009) were calculated using the equations shown, where *z* is the number of validation samples, and *M* is the number of regressors in the model. $\widehat\sigma^2$ is the variance estimator from the likelihood function (Hedderich and Sachs 2018).

9 $$SSR=\sum {\left({m}_{i,x}- {m}_{i,x}^{0}\right)}^{2}$$

10 $$AICc=z\cdot{\log(\widehat\sigma}^2)+2M+2\frac{M(M+1)}{z-M-1}$$

11 $$BIC=z\cdot{\log(\widehat\sigma}^2)+\log(z)\cdot M$$

## Results

### Polymer identification

The peak (Table 2) and glass transition (Table 3) temperatures were determined independently of the regression models. Our results show that DSC is suitable for identifying different polymer types in mixtures within the range of the polymer mass (see Table 1). Table 2 shows the mean peak temperatures and the largest deviations from the mean value for the semi-crystalline polymers (*n* = 30). The mean values of the glass transition temperature and the largest deviations from the mean value of the transition temperature are shown in Table 3. According to Wampfler et al., the glass transition temperature is the mean value between *T*_g,h_ and *T*_g,c_ (Wampfler et al. 2022). These characteristic temperature values are used for the polymer-specific identification of MP in mixtures.

**Table 2:** Identification of semi-crystalline polymers by phase transition temperature. Uncertainty was calculated using Eq. (8)

Polymer	*T*_p,m_ /°C	± Δ*T*_p,m, max_ /K	*T*_p,c_ /°C	± Δ*T*_p,c,max_ /K
LD-PE	109.1	0.7	95.7	2.7
HD-PE	125.3	2.2	113.4	0.6
PP	159.9	1.4	116.5	1.8
PA 6	217.8	5.7	187.5	1.6
PET	239.8	4.6	172.9	8.6
PCL	53.7	1.2	26.0	1.3

**Table 3:** Identification of amorphous polymers by glass transition temperature. Uncertainty was calculated using Eq. (8)

Polymer	*T*_g_ /°C	± *T*_g,max_/K
PS	103.5	6.9
PVC	79.6	1.5

The melting and crystallization peak temperatures show a deviation from their mean value < 2.7 K for all investigated polymers except PET and PA 6. Additionally, the glass transition temperature of PS shows an increased deviation. The increased deviation in the values for PET and PA 6 could be due to the chemical and physical properties of both polymers and will be discussed later.

**Fig. 4:**
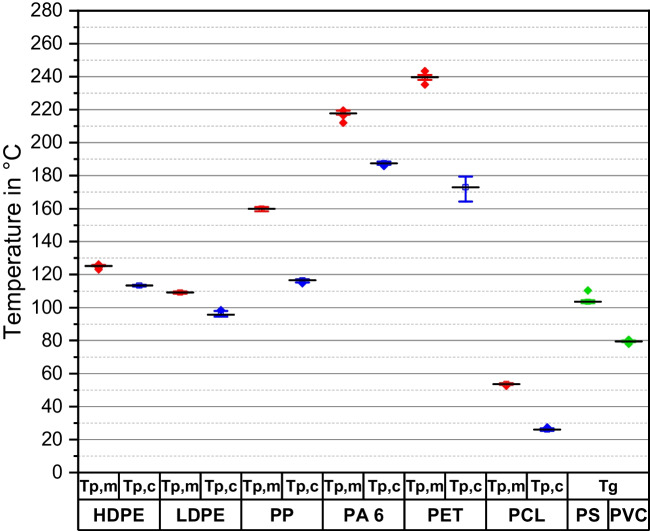
Results of polymer identification by phase transition temperature. The black bars are equal to the mean value (*n* = 30). The red data points represent the melting peak temperatures, and the blue data points represent the crystallization peak temperatures. Additionally, the green data points show the glass transition temperatures of amorphous polymers. A potential superposition was found for PS and both PE types between 100 and 110 °C and for HD-PE and PP between 112 and 118 °C

Figure 4 presents the results of the polymer identification. The majority of the polymers show no superposition of their thermodynamic signals, which could prevent their simultaneous identification in the same sample. However, the possible superposition of the signals from PS and LD-PE during heating and from the crystallization peaks of HD-PE and PP is identified.

### Polymer quantification using DSC

We compared one linear and one multiple regression model, which were the most suitable for the quantification of MP in mixtures. Nevertheless, the 20 model datasets were used to calculate various regression lines, but the two models presented here were the most promising. The “heating” model is a simple linear regression of the mass of the polymer to the melting enthalpy (${\Delta }_{fus}H$). The “multiple” model is a two-factorial regression that takes into account both the melting and crystallization enthalpy. The amorphous polymer data were treated equally.

#### Linear regression

Table 4 shows the results of the “heating” model according to the linear regression using the melting enthalpy or the change in heat capacity during the second heating cycle. For HD-PE, PP, PA6, and PET, the LODs were below 0.1 mg per measurement. It is striking that the LOD and thus the LOQ of semi-crystalline polymers are lower than the analytical limits of amorphous polymers.

**Table 4:** Results of linear regression model to determine slope *b* and intercept *n* of the regression line for polymer quantification by DSC. The values in brackets are the empirical LODs and LOQs, which are presented in detail in the “Multiple regression” section

Polymer	HDPE	LDPE	PP	PA 6	PET	PVC	PS	PCL
$b$	0.0068	0.0117	0.0151	0.0193	0.0348	3.7898	3.0551	0.0182
$n$	0.0187	0.0846	0.0084	0.0344	0.0205	− 0.0718	− 0.0296	0.0307
$LOD \space in \space mg$	0.06	0.15	0.06	0.08	0.05 (0.15)	0.33	0.76 (1.00)	0.25
$LOQ \space in \space mg$	0.18	0.46	0.17	0.25	0.16 (0.25)	0.98	2.27 (2.52)	0.75

#### Multiple regression

Table 5 shows the results of the calculations according to the multiple regression for semi-crystalline and amorphous polymers. The LOD for the polymers was derived from the calculated maximum residuals (${d}_{i,max}$), in the same way as for the linear regression.

**Table 5:** Results of multiple regression model to determine polymer-specific parameters ${a^{\prime}}_{1}$, ${a^{\prime}}_{2}$ and intercept $n^{\prime}$. The values in brackets are the empirical LODs at which the phase transition signal could be distinguished from a blank sample (Fig. 5)

Polymer	HDPE	LDPE	PP	PA 6	PET	PVC	PS	PCL
${a{\prime}}_{1}$	0.0073	0.0014	0.0144	0.0059	0.0181	2.6132	2.2861	− 0.0074
${a{\prime}}_{2}$	0.0005	− 0.0087	− 0.0006	− 0.0121	− 0.0165	1.4786	2.4476	− 0.0243
$n{\prime}$	0.0169	0.0321	0.0083	0.0353	0.0413	− 0.0429	− 0.5241	0.0125
${d}_{max}$	0.07	0.11	0.05	0.06	0.06	0.13	0.61	0.04
$LOD \space in \space mg$	0.07	0.11	0.05	0.06	0.06 (0.09)	0.13	0.61 (1.00)	0.04
$LOQ \space in \space mg$	0.20	0.33	0.16	0.17	0.18 (0.23)	0.40	1.84 (2.22)	0.13

**Fig. 5:**
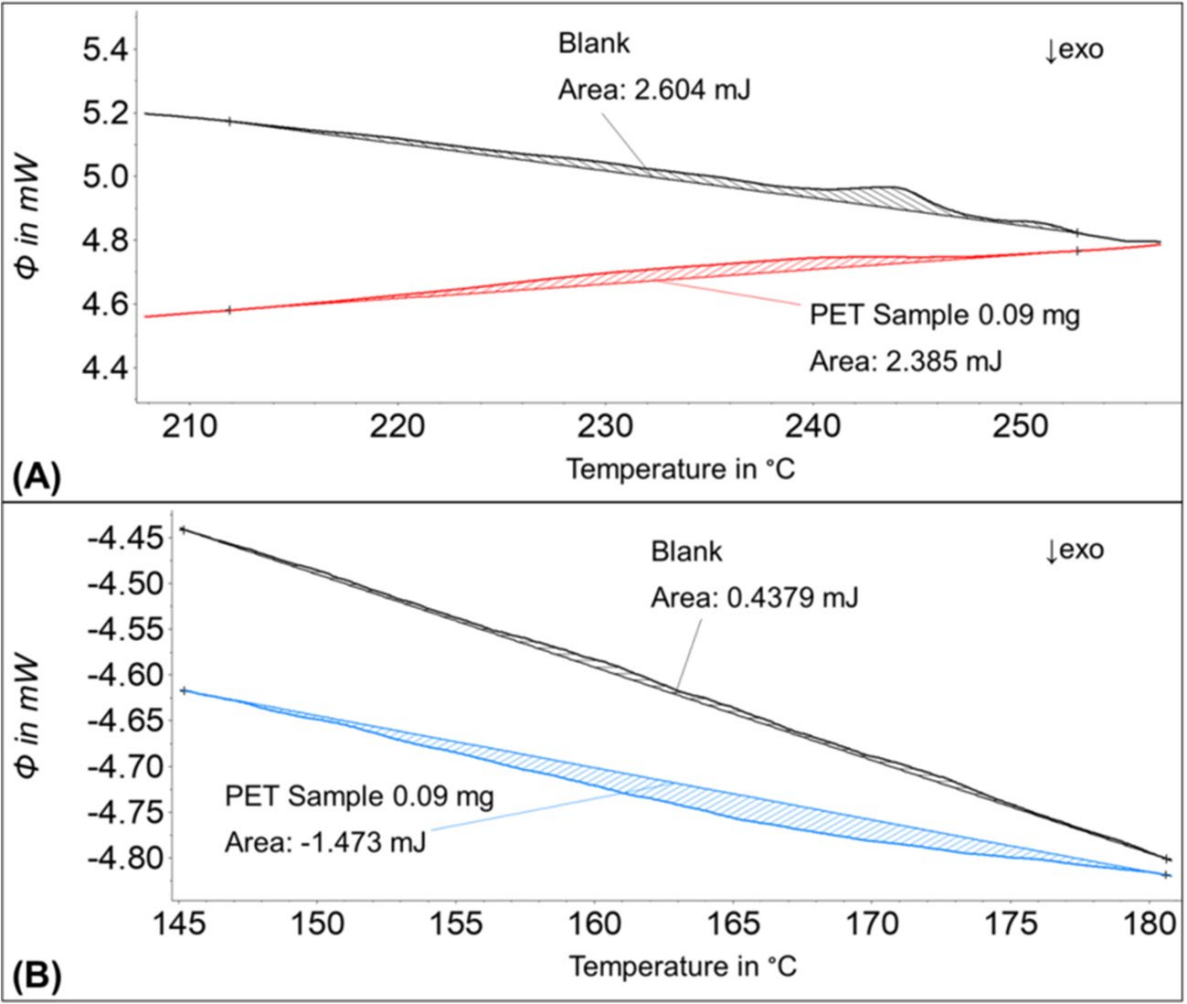
Comparison of a DSC sample containing 0.09 mg PET and sea sand (red and blue) and a blank sample containing only sea sand. **A** The second heating and **B** the cooling curves of both samples

However, we found that the LODs of PET and PS had to be determined differently to obtain reasonable values. According to *d*_i,max_, the postulated LOD for PET would be 0.06 mg per measurement, but, for a sample containing 0.09 mg (0.10 ± 0.05 mg) PET, the value of the melting enthalpy in the melting range of PET was in the same order of magnitude as in the blank samples (Fig. 5A). The crystallization signal at 0.09 mg could already be distinguished from the blank values (Fig. 5B). Consequently, the LOD of PET was set to the polymer mass at which a signal could be evaluated unambiguously in the thermogram during heating *and* cooling. The empirical LOD of PET was set to 0.15 mg per measurement. Additionally, the glass transition signal of PS was too weak to be distinguished from the noise at the predicted LOD of 0.61 mg, and the empirical LOD of PS was set to 1.0 mg per measurement. Consequently, the LOQs of PET and PS were also adjusted. The LOQ can be described according to Eq. (12). The equation does not differ from that used in the previous calculation, but the variable LOD is now larger than ${d}_{i,max}$.

12 $$LOQ=LOD+2\cdot {d}_{i,max}$$

#### Comparison of regression models

**Fig. 6:**
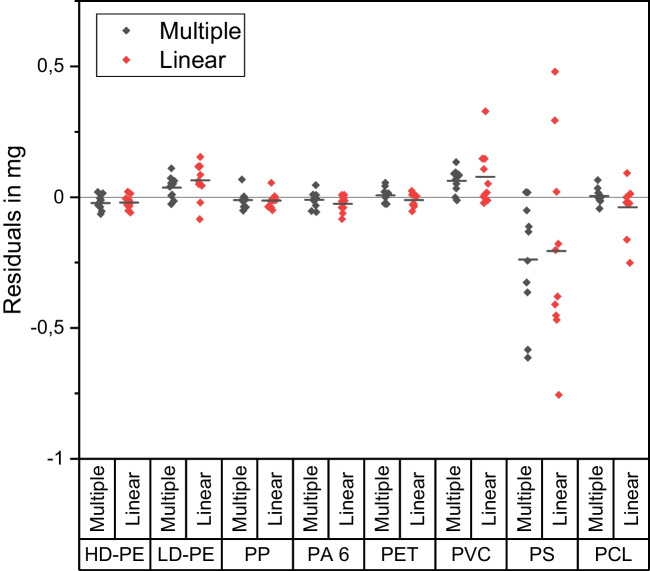
Residuals of predicted polymer mass in validation samples from expected value for different regression models. The expected value equals the actual polymer mass, which was measured during sample preparation

Figure 6 shows the residuals (${d}_{{\text{i}},{\text{x}}}$ and ${d}^{\prime}_{i,x}$) of the validation data from the expected values for the two regression models according to Eqs. 5.1 and 5.2. Comparing the average deviations of the residuals from an optimum value (${d}_{{\text{i}},{\text{x}}}=0$) for the different polymer types, no clear trend can be derived. All regression models provide small residuals in the polymer mass range. However, in all models, the predicted masses of amorphous polymers show a larger deviation from the expected value than for semi-crystalline polymers. Nevertheless, the multiple regression model shows an increase in precision for LD-PE, PCL, and both amorphous polymers by reducing the total range of residuals. This effect is more prominent for the amorphous polymers than for the semi-crystalline polymers.

**Fig. 7:**
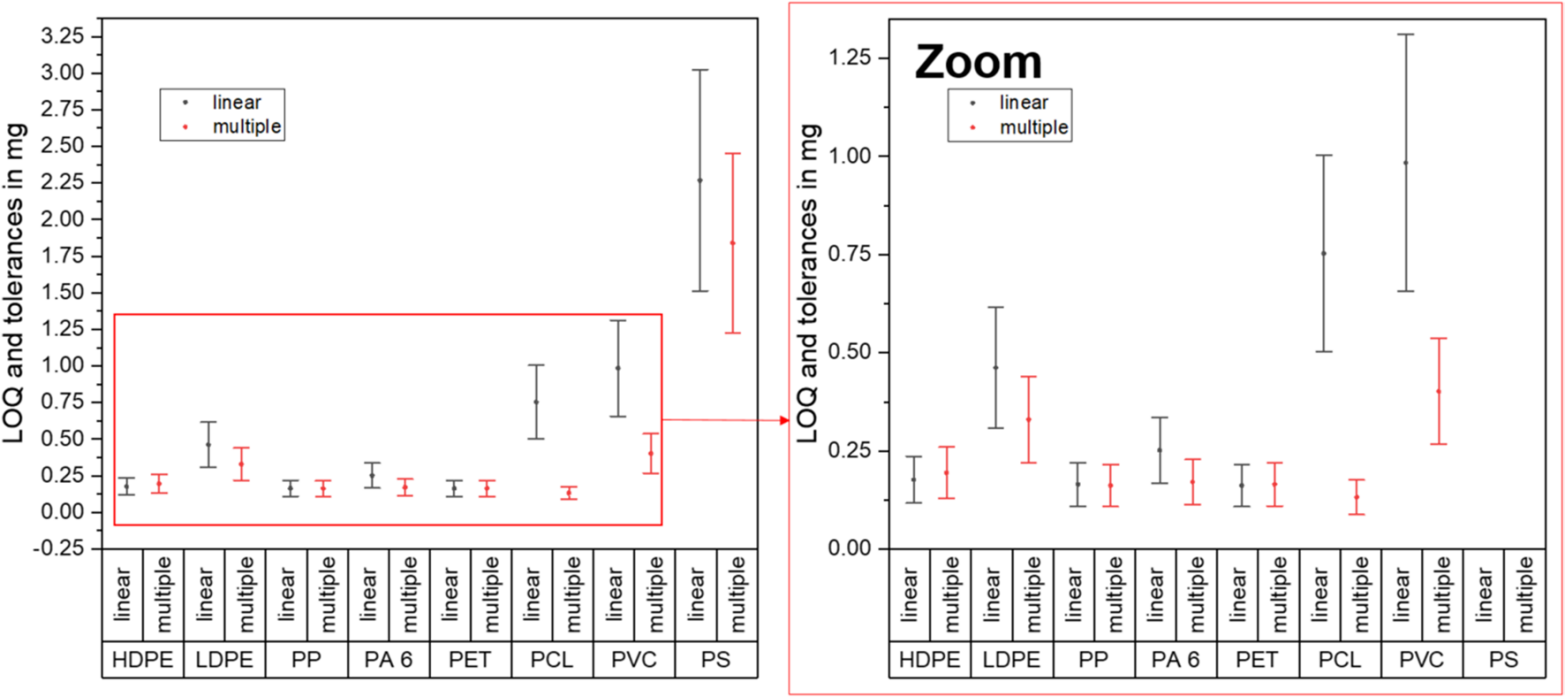
Comparison of analytical limits obtained from linear (“heating”) and multiple regression models. The dots show the LOQs; the error bars are the tolerance levels ($\pm {d}_{i,max}$)

A more practical means to compare the regression models is the comparison of the calculated analytical limits. Figure 7 shows the LOQs of the investigated polymer types for their determination by DSC measurements. The LOQs are shown as dots for the individual polymers, and the tolerance values (${\pm d}_{i,max})$ are shown as error bars. This comparison is carried out for the “heating” and the “multiple” regression models. According to the LOQs, a qualitative result regarding the presence of MP in a sample can be obtained at small polymer masses per measurement. For the semi-crystalline polymers, the LOQ is below 0.25 mg (= 250 µg) or 0.11 mg (= 110 µg) for the linear or multiple regression, respectively. On the other hand, amorphous polymers show a higher LOQ in general, but the multiple regression results in lower values compared to the linear regression.

Moreover, an average LOQ below 0.50 mg per measurement is found for the polymers HD-PE, LD-PE, PP, PA 6, and PET, independently of the regression model. The highest LOQs are found for the amorphous polymers PS and PVC for both regression models. For the remaining polymers, both regression models can be treated equally. Nevertheless, the robustness of the multiple regression is higher due to the increased number of regressors. Especially for PCL and PVC, the multiple regression leads to a smaller tolerance and smaller LOQs. The tolerance of the laboratory balance used is 0.03 mg. This means that no reliable LOD below 0.03 mg can be achieved. The balance used has a considerable influence on the analysis result. At the same time, the very high precision of the DSC analysis at the level of a certified laboratory balance can be assumed.

## Discussion

### Identification of polymeric substances

Our results show that it is possible to identify MP particles in sand mixtures according to their thermodynamic fingerprints. We were able to distinguish eight different polymers within the range of the polymer mass (Tables 2 and 3). The polymer-specific melting or crystallization peak temperatures showed very low uncertainty. Most investigated polymers showed a deviation < 2.7 K in the melting or crystallization peaks. The only exceptions were PET and PA 6. Hence, this parameter seems to be a suitable identification criterion for MP. The low LODs of the investigated polymers (Tables 4 and 5) show that MP contamination can be identified by our method. However, the LODs were polymer-specific and must be determined for each polymer individually.

PET shows broad peaks in the thermogram due to its slow crystallization behavior under the temperature gradient of 20 K/min. The broad peak results in a plateau-like signal, which prevents clear peak determination, because the peak maximum or minimum is set randomly on the plateau. Thus, an increased distribution of the determined peak temperature is obtained. The increased distribution of the peak temperature of PA 6 can be explained by the different morphological crystal structures at a heating rate of 20 K/min. These two structures show overlapping melting signals in the thermogram, leading to a double peak (Wunderlich 2005, S. 660). The first peak belongs to the *γ*-phase and the second peak to the *α*-phase. Depending on the ratio of the two crystal structures, the intensity of the peaks varies and influences the measured peak temperature. Hence, clear peak temperature determination becomes difficult. However, the double peak in the thermogram is very distinctive for PA 6, so this feature can be used as an identification criterion too.

The simultaneous detection of different polymer types in one sample is crucial in the application of DSC for the analysis of MP in environmental samples. Therefore, it is important to identify overlapping signals of different polymer types. The visualization of the results in Fig. 4 shows the possible superposition of the signals of PS and both PE types during heating and of the crystallization peaks of HD-PE and PP. However, the identification of HD-PE in the presence of PP is still possible because of the different melting peak temperatures. Contrastingly, the identification of PS in the presence of any polyethylene would not be possible due to the width of the melting and crystallization peaks, and this requires further research. Nevertheless, one advantage of using DSC is the identification of HD-PE next to LD-PE. These polymers have similar chemical and physical properties, which prevents their simultaneous identification via spectroscopic methods, but the differences in their thermodynamic behavior make their identification by DSC possible.

### Quantification

We were able to prove that DSC can be used to quantify the polymer content in particulate matrices. All investigated regression models resulted in precise regression lines. However, our results did not provide one optimal regression model for all polymers. To evaluate the quality of the regressions, we used the residue of the predicted polymer mass from the actual spiked mass. It was assumed that the optimum model would show the smallest deviation from the expected value for the polymer type. However, we found that the optimum regression model depended strongly on the polymer type. Table 6 shows an evaluation of the regression according to SSR, AICc, and BIC. The parameters for the polymers HD-PE, PP, and PET do not indicate an advantage of multiple regression over linear regression. The assertions of the parameters for model selection are consistent and do not contradict each other. However, the multiple models, evaluated based on the SSR, show no significant differences (HD-PE, PP, and PET) or higher precision (LD-PE, PCL, PA, PVC, PS) than the linear models. Consequently, it is generally possible to use multiple regression.

**Table 6:** Overview of parameters from regression analysis

Polymer	Regressor	SSR	AIC.c	BIC
LD-PE	${\Delta }_{{\text{fus}}}H$	0.088	− 196	− 196
LD-PE	${\Delta }_{{\text{fus}}}H,$ ${\Delta }_{cry}H$	0.029	− 286	− 287
HD-PE	${\Delta }_{{\text{fus}}}H$	0.010	− 265	− 265
HD-PE	${\Delta }_{{\text{fus}}}H,$ ${\Delta }_{cry}H$	0.011	− 261	− 262
PP	${\Delta }_{{\text{fus}}}H$	0.009	− 340	− 340
PP	${\Delta }_{{\text{fus}}}H,$ ${\Delta }_{cry}H$	0.009	− 336	− 337
PA 6	${\Delta }_{{\text{fus}}}H$	0.015	− 305	− 305
PA 6	${\Delta }_{{\text{fus}}}H,$ ${\Delta }_{cry}H$	0.010	− 318	− 319
PCL	${\Delta }_{{\text{fus}}}H$	0.100	− 230	− 230
PCL	${\Delta }_{{\text{fus}}}H,$ ${\Delta }_{cry}H$	0.008	− 275	− 276
PET	${\Delta }_{{\text{fus}}}H$	0.007	− 242	− 242
PET	${\Delta }_{{\text{fus}}}H,$ ${\Delta }_{cry}H$	0.007	− 277	− 279
PVC	${\Delta }_{h}{C}_{p}$	0.166	− 216	− 215
PVC	${\Delta }_{h}{C}_{p}, {\Delta }_{c}{C}_{p}$	0.058	− 229	− 230
PS	${\Delta }_{h}{C}_{p}$	1.695	− 66	− 65
PS	${\Delta }_{h}{C}_{p}, {\Delta }_{c}{C}_{p}$	1.046	− 118	− 119

For the amorphous polymers (PVC and PS), a significant increase in precision and accuracy can be seen. This is shown in the form of a high average residue and a large deviation in the residues for amorphous polymers (Fig. 6). Such large deviations in the residue values are due to the low intensity of the exploited glass transition signal. Glass transitions are considered second-order phase transitions because the signal for a change in heat capacity is based on the change in the internal volume of a polymer. Thus, glass transitions are not classic phase transitions. The intensity of these signals is approximately one hundred times lower than that of the signal of the melting or crystallization of a semi-crystalline polymer of the same mass.

The application of multiple regression can increase the robustness of the model because it depends on two regressors. For PVC and PS, a significant reduction in the residuals in the multiple regression was found. Moreover, for the semi-crystalline polymers, a small improvement in the model output was observed. It becomes clear that multiple regression does not necessarily achieve higher precision and accuracy. However, when using multiple regression, the optimum precision can be obtained across all investigated polymers. Additionally, increasing the number of regressors also increases the capacity for the quantification of one specific polymer type. This is important with respect to potential differences in one polymer type produced by different manufacturers. In the present work, this was demonstrated by the polymer PET. Using the method of multiple regression, the postulated LOQ of 0.19 mg (Bitter and Lackner 2021) was achieved for a very good approximation (0.23 mg, Table 5). It should be noted that the polymers in the mentioned study were of different origin which highlights the robustness and representativeness of the DSC method. Bitter and Lackner (2021) also showed the high robustness of the DSC method against organic material.

The common linear regression, using the melting enthalpy to determine the crystallinity and subsequently the mass of the polymer, shows good results for semi-crystalline polymers. Additionally to the melting enthalpy, the crystallization enthalpy could be used for linear regression. However, a “cooling” model is not proposed because the cooling process, and thus the enthalpy of crystallization could not be calibrated easily due to the supercooling effect (Höhne et al. 2003, p. 84). Due to this effect, the crystallization processes are very specific and would not allow the reliable, direct calibration of the DSC device. The calibration of the crystallization enthalpy would be possible using liquid crystals (Menczel and Prime 2009), but these methods were not applied in this study. Nevertheless, Höhne et al. describe a linear relationship between the crystallization enthalpy and melting enthalpy. Consequently, the calibration of DSC cooling is possible but is subject to adjustment by the calculated correction factors (Höhne et al. 2003, p. 97). Thus, the interpretation of the thermogram of cooling depends on that of heating. For this reason, no individual cooling analysis was carried out. However, the cooling information was included in the multiple model.

Nevertheless, the use of multiple regressions must be critically assessed. A fundamental aspect of multiple regression is that there is no physical model with two independent terms. The melting enthalpy, as well as the crystallization enthalpy, depends on the degree of crystallization. However, the physical processes of melting and crystallization differ during detection using DSC. Hence, the linear factor between the melting and crystallization enthalpies is not constant. The high level of correlation based on their shared physical origin is indicated by a high level of multicollinearity.

The regressors ${\Delta }_{{\text{fus}}}H$ and ${\Delta }_{cry}H$ exhibit a high degree of multicollinearity. This means that both regressors (${\Delta }_{fus}H$, ${\Delta }_{cry}H$) show a strong dependence on the same polymer property, which is the degree of crystallinity. Due to the high degree of multicollinearity, the uncertainty of the coefficients (${a^{\prime}}_{1}$ and ${a^{\prime}}_{2}$) increases. However, the higher uncertainty of the coefficients cannot be identified in our results compared to the linear regressions. The possible application of higher-factorial models with more than two regressors cannot be conclusively assessed at this point. The complexity of the models and the conditional dependence between individual regressors (for example, ${T}_{1,m}$ and ${T}_{2,m}$ to ${\Delta }_{{\text{fus}}}H)$ cannot necessarily be captured with multiple linear models. The coefficient of determination for the multiple regression has to be verified by using polymers from different manufacturers. In addition, a clear and reproducible definition of the integration limits or baselines in the thermograms must be established.

The use of an intercept in Eqs. 1, 2, 3, and 4 must also be discussed in relation to the physically describable basis of the model. Höhne et al. do not describe an intercept in the calculation between mass and heat, but they note that it only applies to ideal parameters. Höhne et al. also show that the linearity between the peak area and mass does not apply to very small masses (Höhne et al. 2003, p. 248). For very small sample masses, the device-specific thermal signal cannot be neglected. The linearity shown in the model used can therefore not be ideally extrapolated to the coordinate origin. For this reason, an intercept was permitted in the models.

In the context of MP analysis in environmental samples, the presented method can be used to determine the MP mass per sample mass or volume. The MP content in sediments from the Elbe River (Germany) was reported to be approximately 0.8 mg/kg PE (Laermanns et al. 2021) to 16 mg/l PE (Scherer et al. 2020). Adomat et al. reported the MP content in river sediments to be between 16 and 1000 mg/kg globally (Adomat 2021). Thus, the LOQs for semi-crystalline polymers are sufficient to determine MP in river sediment samples via DSC. With respect to amorphous polymers, the application of DSC analysis is limited. The high margin of error of 0.6 mg in the specification of the mass of PS in a sample only allows a limited quantitative assessment. However, the identification of PS can be achieved within the investigated range of the polymer mass. The application of more complex systems for the quantitative determination of microplastics in sediments should be considered in subsequent work. The influences of matrix-related effects by sediments should be quantified, as well as their effects on the DSC signals. Heteronucleation by fine sediment components or homonucleation during aging (Menzel et al. 2022), and the correspondingly different crystalline phases, might influence the phase transition behavior and thus the detected heat flow. The existing crystallization models described by Avrami (Wunderlich 2005) or Hoffman–Lauritzen should be discussed for a description or may need to be adapted. Hence, the crystallization process is not isothermal, and a description via the Hoffmann–Lauritzen (Vyazovkin et al. 2005) model would be promising.

Moreover, we provided the regression lines as the absolute mass of the polymer, which could be detected by the method. According to the experimental setup, the data could be given in content units such as mg/kg, with equal results. We suggest calculating the MP content after a measurement. Hence, the power of the presented method depends strongly on the enrichment of MP by the particulate matrix or filtration. Kurzweg et al. investigated a combination of electrostatic separation and DSC for comprehensive MP monitoring in sediments. This study postulated an LOQ of 2.3 mg/kg (Kurzweg et al. 2022). Additionally, the possible impact of sample preparation and matrix influences could be controlled by using an internal standard. In this context, PCL shows great potential as an internal standard due to several aspects. As a biodegradable polymer, high blank values in environmental samples are not expected. Furthermore, the signals of PCL in the thermogram (*T*_p,m,PCL_ = 53.7 ± 1.2 °C, *T*_p,c,PCL_ = 26.0 ± 1.3 °C) do not lead to an overlap with other signals from common plastics (*T*_p,m_ and *T*_p,c_ > 90 °C), and multiple regression can achieve an LOD of 0.07 mg.

Finally, it should be noted that the polymers selected in this study were related to common plastics. No copolymers or elastomers, such as tire abrasion, were included in this study. These polymeric materials have their own characteristic thermograms, which correlate with the individual compositions of the copolymers, the degree of crosslinking (elastomers), and the composition of the additives (tire abrasion). Although these materials are detectable by DSC, they do not fulfill the criteria necessary to identify their polymer types. While a quantitative and qualitative conclusion on the mentioned polymeric materials is not possible, the artificial origin of the materials can be revealed via DSC analysis. Because of the non-destructive nature of the analysis, the samples used in DSC can be subjected to other instrumental methods for microplastic determination, which could facilitate the identification and quantification of the microplastic loads of certain samples. Polymers such as PVC indicate a limitation of the method with regard to non-destructive analysis. The thermal degradation of PVC starts at 150 °C. Consequently, polymers with thermal signals above 150 °C cannot be detected. This limitation, as well as the mutual influence of the thermal signals in the simultaneous determination of polymers in DSC, has already been discussed by Sorolla-Rosario et al. 2022.

The proposed DSC method can considerably reduce the number of samples that need to be analyzed in a complex procedure. Thus, DSC and the thermodynamic fingerprint show high potential with regard to fast and robust MP analysis, with comparatively low investment costs.

## Conclusion

In this study, DSC was evaluated by using regression analysis to enable MP analysis in sediments. The LODs and LOQs of eight common polymers (LD-PE, HD-PE, PP, PA, PET, PCL, PS, and PVC) were determined by using two different regression models. A linear and a multiple regression model were applied in the range of 0.05–1.50 mg per measurement. The multiple regression resulted in increased reliability and robustness in the analysis compared to the linear regression model due to its increased number of regressors. However, the precision of the linear regression models was only slightly weaker. Generally, semi-crystalline polymers can be detected to a better extent than amorphous polymers. The main reason for this is the increased intensity of the melting and crystallization signals compared to the signals of the glass transition. The determined LOQs were 0.20 mg for HDPE, 0.33 mg for LDPE, 0.16 mg for PP, 0.17 mg for PA, 0.23 mg for PET, 0.13 mg for PCL, 0.40 mg for PVC, and 2.22 mg for PS. These analytical limits would be sufficient for MP monitoring in sediments.

However, multiple regression has to be evaluated critically due to the multicollinearity of the two regressors, ${\Delta }_{{\text{fus}}}H$ and ${\Delta }_{{\text{cry}}}H$. Both regressors depend to the same extent on the crystallinity of a polymeric material. Moreover, matrix-related effects such as heteronucleation should be investigated in future work. To improve the robustness of MP analysis against matrix-related effects, we propose the introduction of PCL as an internal standard.

We conclude that the identification and quantification of PE, PP, PA, PET, PCL, PS, and PVC by using DSC has been demonstrated successfully. The use of multiple regression in the qualitative determination of microplastics in sediments increases the robustness of the analytical method. The DSC method is therefore suitable for further studies investigating the transport and fate of microplastics in sediments. Given the possibilities of non-destructive analysis, the presented method can be complemented by further microplastic analysis methods, e.g., thermo-analytical or spectroscopic methods.

## Data Availability

Data is available on request.
